# The therapeutic prospects and challenges of human neural stem cells for the treatment of Alzheimer's Disease

**DOI:** 10.1186/s13619-022-00128-5

**Published:** 2022-09-02

**Authors:** Chunmei Yue, Su Feng, Yingying Chen, Naihe Jing

**Affiliations:** 1 State Key Laboratory of Cell Biology, CAS Center for Excellence in Molecular Cell Science, Shanghai Institute of Biochemistry and Cell Biology, Chinese Academy of Sciences, University of Chinese Academy of Sciences, Shanghai, 200031 China; 2 Department of Biological Sciences, School of Science, Xi’an Jiaotong-Liverpool University, Suzhou, 215000 China; 3 Bioland Laboratory/Guangzhou Laboratory, Guangzhou, 510005 China; 4 CAS Key Laboratory of Regenerative Biology, Guangdong Provincial Key Laboratory of Stem Cell and Regenerative Medicine, Guangzhou Institutes of Biomedicine and Health, Chinese Academy of Sciences, Guangzhou, 510530 China; 5 School of Life Science and Technology, ShanghaiTech University, Shanghai, 201210 China

**Keywords:** Brain disorders, Alzheimer's disease, Stem cell-based replacement therapy, Neural subtype-specific transplantation, Brain region-specific transplantation, Cognitive ability

## Abstract

Alzheimer's disease (AD) is a multifactorial neurodegenerative disorder associated with aging. Due to its insidious onset, protracted progression, and unclear pathogenesis, it is considered one of the most obscure and intractable brain disorders, and currently, there are no effective therapies for it. Convincing evidence indicates that the irreversible decline of cognitive abilities in patients coincides with the deterioration and degeneration of neurons and synapses in the AD brain. Human neural stem cells (NSCs) hold the potential to functionally replace lost neurons, reinforce impaired synaptic networks, and repair the damaged AD brain. They have therefore received extensive attention as a possible source of donor cells for cellular replacement therapies for AD. Here, we review the progress in NSC-based transplantation studies in animal models of AD and assess the therapeutic advantages and challenges of human NSCs as donor cells. We then formulate a promising transplantation approach for the treatment of human AD, which would help to explore the disease-modifying cellular therapeutic strategy for the treatment of human AD.

## Background

As one of the most prevalent neurodegenerative disorders, Alzheimer's disease (AD) worsens with time (Masters et al., 2015). Neuropathologically, AD is characterized by the presence of β-amyloid (Aβ) plaques and neurofibrillary tangles in the brain at the early stage, as well as extensive neuron and synapse loss in the late phase. Clinically, AD is characterized by a progressive decline in the patient’s cognitive abilities, personality, and behavioral abnormalities. In general, the neuropathological changes occur in the brain years, even decades, before clinical symptoms become noticeable in patients (Perlmutter, 2016). Therefore, AD has an insidious onset, followed by a slow and long pre-symptomatic progression. AD is considered to be a multifactorial syndrome associated with aging rather than a single disease (Selkoe, 1999). Despite intensive investigation, the pathogenesis of AD remains largely unclear and has primarily hampered the search for effective drugs or the development of novel therapeutic strategies for the disease. To date, this brain disorder remains incurable.

Mounting evidence indicates that synaptic dysfunction or neuronal loss is induced by diffusible Aβ oligomers at the pre-symptomatic stage. This impairs the integrity of neural circuits in the brain, which directly leads to the cognitive decline of patients (Davies et al., 1987; Palop and Mucke, 2010; Small et al., 2001). The severity of synapse or neuron degeneration is highly correlated with the degree of cognitive decline of AD patients in the late phase (Terry et al., 1991). These studies imply that AD is a result of cumulative synaptic failure over decades (Selkoe, 2002; Sheng et al., 2020). Therefore, treatments that could functionally replace lost neurons and promote the regeneration of damaged synapses show promise for restoring the integrity of neural circuits in the AD brain (Canter et al., 2016).

With the advances in the stem cell field, stem cells have become attractive to the development of potentially powerful therapeutic strategies for different brain disorders (Björklund and Lindvall, 2000; Kiskinis and Eggan, 2010; Lindvall and Kokaia, 2010). Multiple studies have confirmed that neural stem/progenitor cells (NSCs), derived from pluripotent stem cells or reprogrammed from adult somatic cells, could replace lost, dysfunctional, or degenerated neurons in animal models, and might, in turn, reverse the damage caused by disrupted neural circuits in the diseased brain (Fujiwara et al., 2013; Hemmer et al., 2014; Liu et al., 2013; Yue et al., 2015). Compared with NSCs from other species, human NSCs apparently possess unique therapeutic advantages and hence have become well-accepted donor cells in regenerative medicine for various brain disorders, such as Parkinson's disease (PD) (Goldman, 2016). In contrast to the limited brain-region and subtype-specific neuronal loss found in PD, gradual but massive loss of neurons and extensive, severe synaptic degeneration is found in the brains of AD patients. However, it remains uncertain whether grafted neural stem cells could rectify the widespread damage seen in the brains of AD patients as such NSC-based therapies have been performed mainly in mouse models. Thus, there is currently controversy about whether AD is a suitable or medically feasible target for cell replacement therapy.

This review will briefly describe the exploration of stem cell-based therapies in AD animal models, address the progression of cellular replacement studies for AD, and consider the possible mechanism underlying NSC-based therapies. It will also discuss the therapeutic prospects and challenges of human NSCs as donor cells and define the crucial steps towards developing disease-modified cellular therapies for AD.

## The progress of cellular replacement studies in AD animals

With advances in stem cell biology and biotechnology, proof-of-concept studies on stem cell-based replacement therapies have been carried out in animal models of AD (Oliveira and Hodges, 2005; Sugaya and Brannen, 2001). Among these studies, the therapeutic potential of different stem cells has been assessed in animal experiments, including non-neural stem cells, neural cells and NSCs. Mesenchymal stem cells were tested in different AD mice models (Lee et al., 2010a, 2010b). However, the lack of characterization (growth, differentiation, and migration, etc.) of mesenchymal stem cell grafts in the host brain means that there remain uncertainties about the suitability of non-neural stem cells for brain transplantation. Even if grafted non-neural cells have shown some therapeutic impacts in AD mice models, more studies have focused on neural cells, particularly NSCs. After transplantation into the hippocampus of AD mice, cultured astrocytes have been shown to migrate around Aβ plaques and actively clear Aβ cells and internally deposited peptides (Pihlaja et al., 2008). Further, neurosphere derived from mouse embryonic stem cells (ESCs) were reported to generate cholinergic neurons in the cortex of the brain in mice bearing nucleus basalis magnocellularis (NBM) lesions (Wang et al., 2006) (Table 1). Even when the ESC-neurosphere contained more than one type of NSCs, mice with NBM lesions had reduced cholinergic deficits and disruption of their working memories (Wang et al., 2006). Consistently, motor neuronal progenitors with posterior regional identity from mouse ESCs treated by sonic hedgehog (SHH) and retinoic acid (RA) were found to differentiate into motor neurons after they were transplanted into the basal forebrain, which improved the cognitive function of NBM-lesioned rats (Moghadam et al., 2009) (Table 1). Further, it has been demonstrated that expanded murine NSCs rescue the cognitive deficits of AD mice upon hippocampal transplantation (Blurton-Jones et al., 2009).

**Table 1:** The summary of representative NSCs cited in this paper

	**ESC/iPSC-derived NSCs**	**iNPCs**	**Relevant citations**
Morphogens/TFs	ESCs, No morphogen		Wang et al., 2006
	ESCs + SHH + RA		Moghadam et al., 2009
	ESCs + SHH		Liu et al., 2013
	iPSC + RA + Noggin		Fujiwara et al., 2013
	ESCs + SHH + BMP9		Yue et al., 2015
		4 YAMANAKA factors	Zhang et al., 2019
Neuronal subtypes	ChAT neurons		Wang et al., 2006; Moghadam et al., 2009
	BFCNs and GABAergic neurons		Liu et al., 2013
	GABAergic neurons		Fujiwara et al., 2013
	BFCNs and Glutamatergic neurons		Yue et al., 2015
		Glutamatergic neurons	Zhang et al., 2019
Host AD mouse	NBM-lesioned mouse/rat		Wang et al., 2006; Moghadam et al., 2009; Liu et al., 2013
	Transgenic AD mouse	Transgenic AD mouse	Fujiwara et al., 2013; Yue et al., 2015; Zhang et al., 2019
Homogeneity	Heterogenous	Homologous	Liu et al., 2013; Yue et al., 2015; Zhang et al., 2019
Region identity	Hard to be defined	Can be defined	Liu et al., 2013; Yue et al., 2015; Zhang et al.,^ 2019^

Abbreviations: *NPCs* neural stem/progenitor cells, *iNPCs* induced NPCs, *AD* Alzheimer’s disease, *TF* transcription factors, *ChAT* choline acetyltransferase, *BFCNs* basal forebrain cholinergic neurons, *GABA* γ-aminobutyric acid, *SHH* sonic hedgehog, *RA* retinoic acid

With better control of neural fate commitment in vitro, pre-differentiated NSCs with regional identity and higher quality can be cultured from ESCs and induced pluripotent stem cells (iPSCs). This has allowed researchers to attempt to assess the therapeutic potential of ESC/iPSC-derived NSCs, particularly human NSCs, in AD animal models in a more precise manner. For instance, human ESCs gave rise to medial ganglionic eminence (MGE) neural progenitor cells with the treatment of SHH, then the engrafted MGE progenitors were reported to differentiate into the basal forebrain cholinergic neurons (BFCNs) and γ-aminobutyric acid (GABA) interneurons, thereby correcting the learning and memory deficits in mice with a damaged medial septum (Liu et al., 2013) (Table 1). Similarly, human iPSC cultured with SHH, RA, and Noggin differentiate into neural progenitors that have been shown to give rise to cholinergic and GABAergic neurons in the hippocampus of AD mice and rescue spatial memory loss (Fujiwara et al., 2013) (Table 1). Additionally, the progenitors of BFCNs from both human and mouse ESCs treated by SHH and BMP9 give rise primarily to BFCNs after transplantation into NBM and have been reported to markedly ameliorate the cognitive symptoms of transgenic AD mice (Yue et al., 2015) (Table 1). In addition to human NSCs from pluripotent stem cells, self-renewing human NSCs have been derived from the fetal brain by some biotech companies and have been tested as donor cells. Hippocampal transplantation of research-grade human NSCs from Angecon Biotech (Shanghai, China) was found to rescue the cognitive defects of AD mice (Li et al., 2016). Surprisingly, hippocampal transplantation of research-grade human NSCs from Neuralstem Inc. (Germantown, MD) also improved the cognition of AD mice regardless of whether immunosuppression treatment was used post-transplantation (McGinley et al., 2018, 2017). However, some inconsistencies have also been noted, which cast doubt on the therapeutic potential of fetal brain-derived NSCs in the AD mouse model. Human NSCs established by Stem Cells Inc. (Palo Alto, CA) were reported to differentiate into mature neurons in the hippocampus and alleviate the cognitive deficits of AD mice (Ager et al., 2015). Unexpectedly, however, a follow-up study by the same lab reported that the clinical-grade human NSCs from the same company failed to undergo terminal differentiation in the brain of AD mice post-transplantation (Marsh et al., 2017). Even more seriously, grafted NSCs formed ectopic clusters in the lateral ventricle of host AD mice and provided no rescue in cognitive abilities (Marsh et al., 2017). Fortunately, with the discovery and development of direct reprogramming, induced NSCs (iNSC) from terminally differentiated somatic cells offer an attractive alternative option with regard to regenerative therapies (Hemmer et al., 2014). In support of this contention, multipotent iNSCs reprogrammed from human blood cells have been shown to generate glutamatergic neurons and to correct the cognitive deficits of AD mice upon the hippocampal transplantation (Zhang et al., 2019) (Table 1).

In summary, over the past two decades, considerable efforts have been made to investigate stem cell-based replacement therapies for AD in disease models. Further, the therapeutic potential of various cell types has been assessed in different brain regions of AD animals. While the majority of grafted cells displayed therapeutic benefits in an animal model of AD, human NSCs gradually emerged as the most suitable donor cells. Different targeted brain regions have been surveyed, with subsequent studies focusing on those closely associated with cognition, e.g., the hippocampus and basal forebrain. These promising exploration and progression studies suggest that human NSCs might provide unprecedented opportunities to develop innovative treatment strategies for AD (Table 1).

## A possible mechanism underlying functional recovery after NSC transplantation

In general, initial proof-of-concept studies have paid more attention to the therapeutic benefits of grafted neural cells exhibited in AD-like animals but have not provided much insight into the mechanisms underlying functional recovery. For instance, grafted neurosphere or primed neural progenitors were observed to differentiate into type-specific neurons, but the further characterization of such exogenous neurons in the host brain remained unreported (Moghadam et al., 2009; Wang et al., 2006). Once researchers appreciated the unique clinical benefits of NSCs, particularly human NSCs, they started to investigate how they modified the cognitive deficits of AD animals. In summary, the possible mechanisms underlying the action of engrafted NSCs in AD rodents are interpreted as cellular neuroprotection or cellular replacement.

### Grafted NSCs display neuroprotective benefits in AD brain

Grafted mouse NSCs were shown to secrete brain-derived neurotrophic factor (BDNF) that led to increased hippocampal synaptic density and improved hippocampal-dependent cognition of AD mice (Blurton-Jones et al., 2009). Interestingly, BDNF-mediated restoration of cognition does not alter the Aβ or tau pathology in the AD brain, suggesting the action of BDNF through an amyloid-independent mechanism (Blurton-Jones et al., 2009). Consistently, pre-differentiated BFCN progenitors corrected the cognitive deficits of AD mice without changing the global level of Aβ plaques, and this was demonstrated to be due in part to the secretion of BDNF (Yue et al., 2015; Zhang et al., 2019). The neuroprotective effects of grafted NSCs in preventing neuronal degeneration or atrophy, as well as reversing synapse loss, were similar to the results obtained when BDNF was directly administered to brains associated with AD (Nagahara et al., 2013, 2009). In addition, grafted BFCN progenitors secreted acetylcholine and produced acetylcholinesterase in the basal forebrain of AD mice, which were essential for cognitive recovery in AD mice (Yue et al., 2015). These data indicate that NSC-derived neurons possess similar functions to their counterparts in vivo with regard to the metabolism of acetylcholine in the AD brain (Yue et al., 2015). Thus, the neuroprotective benefits of grafted NSCs or derived neurons largely seem to be achieved through the secretion of neurotrophin or neurotransmitters, which might, in turn, contribute to the repair of brain damage and correction of cognitive deficits in AD patients.

### Grafted NSCs function via cell replacement in AD brain

Structural integrity and proper synaptic activities are required for normal brain function. With further investigation of grafted NSCs and derived neurons, it has become clearer that grafted NSCs can functionally replace the degenerated neurons and act through a cell replacement mechanism in addition to their neuroprotective effects on the AD brain. Multiple studies have shown that grafted NSCs display long-term survival and terminal neural differentiation in AD rodents. This supports the contention that NSC grafts are well-tolerated in the pathological environment of the AD brain (Fujiwara et al., 2013; Hemmer et al., 2014; Liu et al., 2013; Yue et al., 2015). Additionally, neurons from grafted human medial ganglionic eminence–like progenitor cells differentiated were found to fire an action potential and express K^+^, Na^+^, and spontaneous postsynaptic currents in lesioned AD mice, indicating that human NSC-derived neurons possess membrane properties that are typical of mature neurons (Liu et al., 2013). Recently, a couple of studies have systematically characterized grafted NSC-derived neurons in terms of their survival, proliferation, differentiation, migration, projection, and integration in AD mice, thereby attempting to assess the potential for NSCs to replace lost neurons and degenerated synapses (Yue et al., 2015; Zhang et al., 2019). The grafted BFCN progenitors were confirmed to give rise to mature BFCNs that display projections and migration patterns that were typical of native BFCNs in the basal nucleus of AD mice (Yue et al., 2015). Elaborate dendritic arbors and long axons arising from the grafted BFCNs were detected, and synaptic structures typically seen between the exogenous and endogenous neurons were observed by electron microscopy (Yue et al., 2015). More importantly, the majority of grafted BFCNs were found to exhibit excitatory and inhibitory synaptic activities, suggesting that they might functionally integrate into the endogenous cholinergic circuitry system in the basal forebrain of AD mice (Yue et al., 2015). A follow-up study from the same lab reported that human iNSCs differentiated to glutamatergic neurons one month after hippocampal transplantation in AD mice (Zhang et al., 2019). The glutamatergic neuronal grafts displayed long-term survival for up to 12 months and stayed healthy without invasion of Aβ plaques or activated microglia (Zhang et al., 2019). An optogenetic assay confirmed that exogenous neurons formed graft-host synaptic connections with endogenous hippocampal neurons and displayed proper postsynaptic activities. This directly demonstrates the functional integration of grafted human neurons into the synaptic networks of the host hippocampus (Zhang et al., 2019). Additionally, the elevated synaptic transmissions reinforced the local neural circuitry of the AD brain, which increased the level of long-term potentiation (LTP), enhanced hippocampal plasticity and, eventually alleviated the cognitive deficits of AD (Zhang et al., 2019). These observations strongly suggest that human NSCs can functionally integrate into the neural networks, replace damaged neurons and strengthen impaired synaptic circuits of the AD brain.

## Clinical prospects for developing a human NSC-based replacement therapeutic strategy for the treatment of AD

**Fig. 1:**
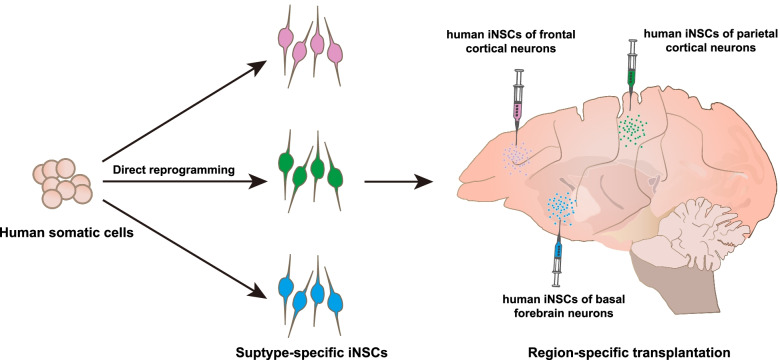
The workflow of postulated subtype- and region-specific cellular replacement therapy for AD. The ideal donor cells will be human induced NSCs (iNSCs) with regional identity that could be generated from adult somatic cells, such as mononuclear cells in peripheral blood, via direct reprogramming. Then, human iNSCs with multiple potential to give rise to subtype-specific neurons could serve as donor cells and will be simultaneously transplanted into brain regions where the same neuron subtypes had been lost or had degenerated. Such subtype- and region-specific transplantations might allow the local and regional replacement of lost neurons, which should consequently lead to the specific and efficient repair of the widely damage neural circuitry AD brain

From a clinical perspective, the development of stem cell-based therapies for AD is still in its infancy (Lindvall and Kokaia, 2005). To achieve the functional replacement of massive neuron loss in multiple brain regions of AD patients, we previously proposed a subtype- and region-specific therapeutic strategy as the disease-modifying therapy for AD (Fig. 1). To this end, it is necessary to develop appropriate human NSCs suitable for transplantation, suitable transplantation strategies, and relevant AD animal models for rigorous assessment of the therapeutic effects of grafted NSCs.

### Generating subtype-specific human NSCs as donor cells

Because mature neurons cannot regenerate and adult NSCs possess a limited capacity to produce new neurons, the human brain displays a limited ability for self-repair in response to injury or disease. Therefore, human NSCs that can be stably generated in vitro would offer a unique and reliable cell resource for the development of disease-modifying therapies. Since diverse subtypes of neurons are damaged and lost as well as multiple brain regions in AD patients, ideally, it is necessary to generate multiple types of human NSCs that each possesses distinct regional identities and the capacity to differentiate into one subtype-specific neuron, and in particular cortical glutamatergic, GABAergic and BFCNs that play essential roles in cognition (Fig. 1).

Early studies showed that mouse ESC-derived medial ganglionic eminence-like progenitors differentiated into almost equal numbers of BFCNs and GABAergic neurons in lesioned AD-like mice (Liu et al., 2013; Yue et al., 2015). In contrast, ESC-derived BFCN progenitors mainly gave rise to BFCNs but with a small proportion of GABAergic and Glutamatergic neurons in the basal forebrain of AD mice (Liu et al., 2013; Yue et al., 2015). These observations indicated that human ESC-derived NSCs were usually generated as complex cell populations with high heterogeneities. Other than that, the ESC-derived NSCs hardly expanded in vitro. Therefore, to obtain sufficient NSCs for transplantation, multiple rounds of neural differentiation from ESCs would have to be carried out to obtain a sufficiently large number of pre-differentiated NSCs. This, in turn, would cause batch variation and affect the reproducibility of cellular treatment. These limitations largely hampered the application of ESC-NSCs in cellular replacement studies for AD.

To produce human NSCs with the consistency and homogeneity necessary for cellular replacement, researchers have tried to directly isolate and expand human central nervous system stem cells (hCNS-SCs) from the fetal brain tissue using cell surface markers (specifically: CD133^+^, 5E12^+^, CD34^−^ and CD45^−^) (Uchida et al., 2000). Transplantation of hCNS-SCs into the lateral ventricles of immunodeficient neonatal mice resulted in proliferation, migration, neural differentiation, long-distance projection, long-term survival, and specific engraftment in numerous regions of the host brain (Uchida et al., 2000). Similar results were observed by transplanting isolated human fetal neural progenitor cells into neurogenic brain regions of adult rats, such as the subventricular zone and hippocampus (Englund et al., 2002a, 2002b; Fricker et al., 1999). However, due to ethical constraints and assessment limitations, fetal brain-derived human NSCs have been considered impractical even though they display great therapeutic potential. Therefore, an alternative approach is required.

Over the past decade, achievements in direct reprogramming have provided genuine opportunities to generate unlimited and lineage-specific human NSCs. The ectopic expression of defined transcription factors with or without small molecules has successfully converted human somatic cells, including fibroblasts (Kumar et al., 2012; Lu et al., 2013; Ring et al., 2012; Yu et al., 2015; Zhu et al., 2014), urine cells (Cheng et al., 2014), mononuclear cells from both cord blood (Bruzos-Cidon et al., 2016; Liao et al., 2015; Tang et al., 2016) and adult peripheral blood (Dowey et al., 2012; Zhang et al., 2019), into iNSCs. The iNSCs have been shown to capture the key features of adult NSCs in the human brain, indicating that they might possess a therapeutic potential that rivals adult NSCs. iNSCs from adult peripheral blood cells were confirmed to differentiate preferentially into cortical glutamatergic neurons both in the dish and in the mouse brain (Zhang et al., 2019). More excitingly, comprehensive assessments show that these human iNSCs exhibit therapeutic benefits in AD mice (Zhang et al., 2019).

Generating human iNSCs that could serve as ideal donor cells is rather challenging. It is difficult to create multiple lines of subtype-specific human NSCs such that each line could differentiate into a given neuron type on demand so as to replace the lost neuron subtypes to a large extent in the AD brain. So far, the reported studies have demonstrated that the generated human iNSCs are found to differentiate predominantly into cortical glutamatergic neurons in a default way (Dowey et al., 2012; Zhang et al., 2019). However, new approaches are needed to create other subtype-specific human iNSCs. In addition, it remains largely uncertain whether human iNSCs behave in vivo in a controlled manner in terms of their survival, proliferation, terminal and matured differentiation, proper migration, and projection, as well as their functional integration. Although the capacities of ESC-NSCs or human iNSCs in survival and differentiation are comparable to those of fetal brain-derived NSCs (Zhang et al., 2019), the latter display wide-scale migration or long-distance projection in the AD brain, while the observations of ESC-NSCs, human iNSCs, or their derivatives do not (Li et al., 2016). It is worth noting that these limitations are likely to compromise the therapeutic efficacy of grafted human iNSCs in the AD brain, which should be addressed in future studies.

### Region-specific transplantation of human NSCs into brain regions vulnerable to AD

Region-specific transplantation strategies have been pursued to cover the many brain regions affected in AD. As shown in Fig. 1, the critical requirement of this approach is to simultaneously and precisely deliver several subtype-specific human NSCs into different brain regions where the equivalent neuron subtypes have been lost. Theoretically, brain regions matching the identities of NSC-derived neurons would be the most suitable niche for the survival, differentiation, and migration of grafted human NSCs. The expectation is that grafted NSCs would differentiate into mature neurons in the specific regions of the AD brain, replace their severely degenerated or lost counterparts in a local and subtype-specific manner, reconstruct a specific neuronal population and re-establish reciprocal connectivity within the host brain. In short, local and regional replacement of lost neurons would specifically target the widely damaged neural circuitry of the AD brain. Re-establishing a close-to-normal neuronal innervation in the cognition-associated brain regions would be the final goal in the quest for a treatment for AD. To achieve the functional replacement of global neuronal loss or to partially replace the major neuron subtypes associated with cognition in AD brain, the types of human NSCs, the dosage, and the targeted brain regions all need to be carefully considered.

Moreover, safely and efficiently delivering human NSCs into the AD brain is obviously of critical importance for successful therapeutic intervention in patients. There are several delivery routes that have been used to inject NSCs into the brains of AD animals, such as intraparenchymal (Liu et al., 2013; Yue et al., 2015), intranasal (Danielyan et al., 2014; van Velthoven et al., 2010) and intraventricular (Hemmer et al., 2014) delivery. Among them, the image-guided cerebral intraparenchymal injection has been the most widely used delivery method, which allows precisely targeting of particular brain regions associated with AD. Thanks to the efforts of many labs, the procedures for intraparenchymal injection have been progressively improved in terms of the injection speed, injection frequency, single or multiple injection sites, and the dosage of donor cells. Currently, the intraparenchymal injection might be the most suitable delivery method for region-specific transplantation of human NSCs for AD. However, intranasal delivery of donor cells into the brain has been shown to be reliable, manageable, and less invasive than widely used surgical transplantation and has been successfully applied to administer a range of donor cells into various disease models (Danielyan et al., 2014; van Velthoven et al., 2010). Thus, intranasal delivery offers an attractive alternative for the introduction of human NSCs into the brains of AD patients. From a clinical viewpoint, the procedures required for surgery-based human NSCs transplantation are far from optimal. Further modification of the cell transplantation process will be needed to develop an effective human NSC-based replacement therapy for AD.

The above achievements and concerns suggest that the subtype specificities are a straightforward requirement when using human NSCs as specialized donor cells for AD. As indicated above, a region-specific transplantation approach is likely to be needed for the simultaneous repair of multiple regions in the AD-affected brain. This implies that donor cells and proper delivery methods need to be optimized to develop an effective subtype- and region-specific cellular replacement strategy as a disease-modifying treatment for AD (Fig. 1).

### Finding an appropriate animal model of AD

Before clinical applications can be envisaged, human NSCs need to be carefully studied in AD-relevant models. It follows that appropriate animal models that allow us to predict the accurate therapeutic benefits of human NSCs are essential prerequisites for the assessment of the reliability and validity of preclinical studies and the success of subsequent translational studies. Currently, AD mouse models are the most widely used animal models in assessing the therapeutic potential of human NSCs because of the easy accessibility. In addition, the handling of AD mice is easy referring to the surgery of cell transplantation and behavioral tests. Unfortunately, the various AD mice used have failed to fully and faithfully recapitulate the pathological features and progression of AD due to the fundamental species-specific differences between humans and rodents. (Drummond and Wisniewski, 2017; LaFerla and Green, 2012). In particular, neuronal loss seldom occurs in currently available AD mouse models, which compromises their usefulness in the evaluation of the efficacy of grafted cells. The lack of reliable animal models has largely hampered the development of disease-modifying therapeutic strategies for AD.

The non-human primate PD models have displayed promising perspectives in developing cell replacement therapies for this disease (Kikuchi et al., 2017; Morizane et al., 2013). Following convincing preclinical data from a PD monkey model, clinical trials of cell replacement therapy for PD have begun. Currently, the successful application of a personalized cell-therapy strategy using autologous, iPSC-derived dopaminergic progenitor cells in a 69-year-old PD patient has been reported (Schweitzer et al., 2020). The clinical and imaging measurements clearly showed that PD symptoms stabilized or improved at 18 and 24 months after implantation. Accordingly, the generation of non-human primate models of AD, closely resembling the human disease, might be helpful to develop disease-modifying cell replacement strategies for better intervention or sustained symptomatic relief in AD patients. Different labs have made efforts to develop a disease model in non-human primates for AD for years. However, a well-established AD monkey model has not been reported yet. Soluble Aβ oligomers (AβOs) have been shown to induce AD-like features in the brains of non-human primates. For example, hyperphosphorylated Tau and the loss of dendritic spines have been observed (Beckman et al., 2019; Forny-Germano et al., 2014)  suggesting that AβOs might be used to generate AD disease models in monkeys. Indeed, considerable progress has been achieved recently.

Thus, it has been reported that repeated AβO injections into the cerebral parenchyma rapidly caused massive Aβ plaques, evident neurofibrillary tangles, profound neuroinflammation, as well as selective neurodegeneration in adult cynomolgus monkeys. Collectively, this indicates that the progression of AD, at least the classical neuropathological features of patient at early phase, were reproduced in non-human primates (Yue et al., 2021). These results suggest that it will be possible to generate an appropriate monkey model for AD upon the administration of AβOs, which could serve as a promising research tool for developing disease-modifying cell replacement therapies for AD.

## Conclusions

Taken as a whole, the findings of transplantation studies using human NSCs in AD animals are encouraging. However, to develop subtype- and region-specific cellular replacement therapies as disease-modifying treatments for AD, extensive studies are required to cope with the challenges associated with generating ideal donor cells and appropriate disease models. As aforementioned, the most promising donor cells should be human iNSCs with subtype specificity and brain-region identity. To obtain subtype- and region-specific human iNSCs, direct reprogramming assays need to be modified and developed to precisely capture the target cells during cell fate conversion, and elaborate culturing systems need to be formulated to consistently maintain the captured cells in the dish. The most suitable disease models should be non-human primate models that could authentically recapitulate both neuropathological features and cognitive deficits of AD patients. Based on the new progress in the development of AD monkeys (Yue et al., 2021), great efforts need to be made to trigger the extensive neuronal loss in the brain of AβO-treated monkey. Then, robust behavioral tasks and platforms that can assess the cognitive abilities need to be established. With donor cells and animal models, convincing data needs to be collected preclinically in AD animal models to demonstrate efficacy and reveal the mechanism(s) underlying any observed functional recovery upon the combined transplantation of human NSCs prior to clinical trials. Undoubtedly, fundamental differences between species will cause discrepancies between AD animals and AD patients with regard to the therapeutic impacts of grafted human NSCs. Given that, pilot studies should be carried out to explore and evaluate the possible challenges for translational studies, in particular the dosage of donor cells, proper delivery strategies, and the immunosuppression regimen. The dosage of human NSCs for AD animals will be different from those for AD patients. Then, the proper number or volume of donor cells needs to be determined in pilot studies, which will help avoid the overgrowth of grafts in the brain of patients. The immunosuppression regimen is critical to the survival of grated human NSCs and raises obvious safety concerns about stem cell-based replacement therapies. Therefore, proper immunosuppression regimens suitable for AD patients must be carefully designed. Even though the development and application of stem cell-based therapies for AD are still at an early stage from a clinical perspective, we believe these efforts will significantly facilitate the transition from proof-of-concept in AD animal studies to human clinical trials, which should eventually offer a meaningful clinical benefit for patients.

## Data Availability

Not applicable.

## Abbreviations

AD: Alzheimer's disease
Aβ: β-Amyloid
AβOs: Aβ oligomers
NSCs: Neural stem/progenitor cells
iNSC: Induced NSCs
PD: Parkinson's disease
ESCs: Embryonic stem cells
NBM: Nucleus basalis magnocellularis
BFCNs: Basal forebrain cholinergic neurons
GABA: γ-Aminobutyric acid
iPSCs: Induced PSCs
BDNF: Brain-derived neurotrophic factor
LTP: Long-term potentiation
hCNS-SCs: Human central nervous system stem cells
MGE: Medial ganglionic eminence
SHH: Sonic hedgehog
RA: Retinoic acid
